# Lyrics do matter: how “coping songs” relate to well-being goals. The COVID pandemic case

**DOI:** 10.3389/fpsyg.2024.1431741

**Published:** 2024-12-23

**Authors:** Adi Levy, Roni Granot, Renana Peres

**Affiliations:** ^1^The Hebrew University Business School, The Hebrew University, Jerusalem, Israel; ^2^Musicology Department, The Hebrew University of Jerusalem, Jerusalem, Israel

**Keywords:** songs, well-being, lyrics, emotion regulation, topic modeling, acoustic features analysis, COVID-19

## Abstract

**Introduction:**

In stressful times, people often listen to “coping songs” that help them reach emotional well-being goals. This paper is a first attempt to map the connection between an individual’s well-being goals and their chosen coping song.

**Methods:**

We assembled a large-scale dataset of 2,804 coping songs chosen by individuals from 11 countries during COVID-19 lockdown. Individuals reported their well-being goals and also named their coping song. We applied an unsupervised topic-modeling approach to identify 15 self-emerging topics from the song lyrics, and connected them to well-being goals.

**Results:**

We found significant association between certain lyrics’ topics and specific well-being goals. This association weakened for participants for which music is highly important. No significant patterns were found for the songs’ acoustic features.

**Discussion:**

This paper posits that song lyrics, despite their brevity and presumed simplicity, can be meaningful for self-regulation of emotional states, and should receive more attention by researchers and streaming services alike.

## Introduction

### Coping songs to achieve mood regulation goals

Listening to music is a simple yet powerful way of coping with stressful situations (e.g., Gabrielsson, 2001; Juslin et al., 2008; Vidas et al., 2021). Music can help regulate one’s stress levels (Baltazar et al., 2019; De Witte et al., 2020) and anxiety (Knight and Rickard, 2001), and help manage negative and painful feelings (Saarikallio and Erkkilä, 2007). In stressful times, people may relate to “coping songs,” or musical pieces that they listen to regularly and that help them through their hardship (Grocke and Wigram, 2006; Silverman, 2009; Hamilton et al., 2013; Vidas et al., 2021). Thus, adopting a coping song can be viewed as a self-regulation praxis that enhances well-being (McFerran and Saarikallio, 2014).

What characterizes the choice of coping songs during stressful times? Do their lyrics share common themes? Is there a distinct musical composition? Or perhaps a certain music-lyrics combination? Is there a link between one’s desire for mood regulation and the chosen song? This paper aims to shed light on these questions.

On the theoretical level, mood regulation involves consciously or unconsciously, setting goals aiming towards a desired emotional state (e.g., venting stress or anxiety, diversion from a current difficulty, enhancing enjoyment, reducing loneliness), and then selecting a musical piece that aids in achieving these goals (Baltazar and Saarikallio, 2019). This paper primarily examines the link between these goals and the chosen coping song.

Considerable research has examined various ways in which music supports emotional regulation and well-being goals (e.g., MacDonald et al., 2013; Baltazar and Saarikallio, 2016). However, the specific connection between self-selected coping songs–including both their music and lyrics- and emotional regulation goals remains largely unexplored. In addition, only a limited number of studies have examined mood regulation mechanisms associated with song lyrics (Hanser et al., 2016; ter Bogt et al., 2017; Baltazar and Saarikallio, 2019; Baltazar and Västfjäll, 2020). This gap is surprising, as songs are the most common form of music that individuals listen to.^1^

We study the link between emotional regulation goals and the chosen coping song in a real-life setting by using a large-scale unique dataset containing 2,804 songs chosen by 5,619 participants in 11 countries following the first COVID-19 pandemic lockdown. We analyze this large data set employing unsupervised topic-modeling methods that enable us to extract themes in the songs’ lyrics, that we then connect to the participants self-reported emotional goals.

Our analysis shows a significant association between certain lyrics topics and specific well-being goals. We found that this association weakens—but is still significant—for participants who indicated that music is highly important in their lives. We did not identify significant relationships between goals and the nominated songs’ acoustic features.

Our contribution is significant in substantive, methodological, and practical terms. Substantially, this is a novel attempt to link the choice of coping songs to mood-regulation goals in a large-scale, real-life setting. We suggest that song lyrics, despite their brevity and presumed simplicity, can be meaningful for listeners. Methodologically, this study is pioneering in linking such an extensive, self-selected collection of coping songs—diverse in language, culture, genre, and epoch—with well-being goals in an ecologically valid yet relatively controlled setting. We also demonstrate the application of unsupervised text analysis techniques to large-scale song data to uncover thematic elements in lyrics. Our findings underscore the importance of incorporating song lyrics in music and well-being research, and have practical implications for streaming services that seek to align songs with listeners’ needs. For streaming platforms, this could mean applying our results within recommendation algorithms to create personalized playlists. By using listening data—or even by directly engaging users—platforms can identify users’ emotional states and well-being goals, and integrate our procedure and results to analyze lyrical themes to recommend songs that more accurately support mood regulation goals.

## Literature review

Predicting which music could assist a given individual to obtain a chosen goal is challenging due to the lack of a complete model of goals and music choice strategy. There are often a number of routes to obtaining the same goal, including blends of various strategies (Gross, 2015). Also, music choice depends on contextual and individual traits such as age (Groarke and Hogan, 2019), gender (Carlson et al., 2015; ter Bogt et al., 2017), personality traits (Garrido et al., 2015), and level of musical sophistication (Carvalho et al., 2022).

Although predicting which musical piece an individual will choose for a specific mood regulation goal is challenging, significant progress has been made in understanding how people use music to regulate their mood or emotions. Studies have shown that music is used for reducing stress and anxiety (De Witte et al., 2020), for diversion, pleasure, elevating and maintaining good mood (Randall et al., 2014; Cook et al., 2019), for solace, release from negative emotions, and repair through reappraisal (Hanser et al., 2016), and for reducing feelings of loneliness through reconnecting to positive nostalgic memories, or surrogating relationships (McFerran and Saarikallio, 2014; Schäfer et al., 2020).

### The role of lyrics in mood regulation

The personal “meaning” of a song for an individual arises from a complex web of interactions between its text, musical composition and performance, the ideas it conveys, and the very personal interpretation and emotions the song evokes in the listener. Within this web, lyrics can potentially play an important role. Yet popular song lyrics have mainly been studied from a socio-cultural perspective as indicators of changes in public sentiment and the relative importance of various social themes such as inequality, hardships, sexuality, violence, or substance use (e.g., Zullow, 1991; Brand et al., 2019; Christenson et al., 2019; Qiu et al., 2019). Other studies have reported analysis of song lyrics (with or without acoustic features) for genre or emotion identification (Logan et al., 2004; Han et al., 2022). Many of these studies use variations of word counting procedures, although usage of more sophisticated topic modeling methods such as Latent Dirichlet Allocation (LDA) is becoming more prevalent (Kleedorfer et al., 2008; Sasaki et al., 2014; Fell et al., 2019, 2022; Nakai and Itoh, 2022).

Those studies that have explored lyrics as a possible aid for personal mood or affect regulation through songs, suggest that listeners can use the message in the lyrics to work through their emotions and possibly to see these emotions in a new light as reflected for example in empowering lyrics (Baltazar and Saarikallio, 2019; Baltazar and Västfjäll, 2020). Alternatively, those in a sad or distressing situation may find solace in sad songs with which they can identify. Here the lyrics can provide a sense of acceptance and belonging (“the artist that sings the songs feels what I am feeling” - Hanser et al., 2016; ter Bogt et al., 2017).

Other studies have sought to examine the relative contribution of the music versus the lyrics in the emotions perceived in the songs. While some found that lyrics influence mood more than music (Stratton and Zalanowski, 1994), others have shown the opposite (Sousou, 1997) with a moderating effect of the listener’s culture (Barradas and Sakka, 2022). The relative role of lyrics depends upon the song’s valence, i.e., lyrics were found to be important in enhancing emotions for songs with sad and angry music, but not in songs whose music was associated with happiness or calm (Ali and Peynircioğlu, 2006; Baltazar and Västfjäll, 2020).

In this paper, we inspect lyrics through the perspective of coping songs and their relationship to mood self-regulation. We focus on the individual level (rather than the socio-cultural), and quantify the connection between the lyrics of the selected songs and self-regulation goals. By analyzing a large number of songs, we can identify common themes in the lyrics and link them to well-being goals.

### The COVID pandemic as a unique opportunity to study how songs help in mood regulation

The COVID-19 pandemic has provided a unique opportunity to explore, in a real setting, the link between coping songs and mood regulation across a large number of songs and individuals. The stress, anxiety, and loneliness experienced by millions, called clearly for implementation of emotion regulation strategies, wherein music listening served an important function (Carlson et al., 2015, 2021; Granot et al., 2021; Hennessy et al., 2021; Krause et al., 2021; Martín et al., 2021; Ziv and Hollander-Shabtai, 2021).

Research has shown that music did indeed help many people to cope with the negative emotions provoked by the COVID-19 crisis (Cabedo-Mas et al., 2021; Carlson et al., 2021; Krause et al., 2021; Martín et al., 2021; Ziv and Hollander-Shabtai, 2021), especially those experiencing high distress (Hennessy et al., 2021). This has been found to hold cross-culturally (Granot et al., 2021; Hennessy et al., 2021). Yet, the size of the positive effect varied as a function of individual differences including age (Ferreri et al., 2021; Granot et al., 2021; Henry et al., 2021; Martín et al., 2021), importance of music in one’s life (Granot et al., 2021; Henry et al., 2021), musical training (Martínez-Castilla et al., 2021) and personality traits like openness to experience (Granot et al., 2021), empathy (Hennessy et al., 2021), and sensitivity to musical reward (Mas-Herrero et al., 2021). The mood regulation strategies found useful are consistent with Baltazar and Saarikallio’s (2019) proposed model, and include reappraisal and positive reframing (Hennessy et al., 2021; Henry et al., 2021), discharge of negative emotions, especially in those higher in anxiety (Hennessy et al., 2021), and seeking out positive/happy music (Ferreri et al., 2021). Yet, none of these studies looked at specific real-time musical selections that supported these coping strategies. One of the few studies that did so examined acoustic features of self-selected songs that helped to “manage stress” among Australian students (Vidas et al., 2021). They found that the 271 songs in their data set had lower energy and valence, and tended to be more instrumental, compared to a benchmark dataset of Howlin and Rooney (2021), which contains 153 songs self-selected to reduce pain. They also found no significant correlations between acoustic/musical features such as energy, valence, tempo, danceability and acousticness, and well-being measures. However, the paper does not address songs’ lyrics.

Our study extends the literature by examining how individuals choose songs to cope with stressful situations. We specifically address both song lyrics and acoustic features, directly investigating the link between the selection of coping songs and mood-regulation goals—an area that has received limited attention. Utilizing a unique dataset of self-selected coping songs and individually rated emotional regulation goals, we explore, on a large scale and in a real-life context, whether the musical composition and lyrics of these songs can shed light on how people use them to cope during challenging times.

## Methods

### Data collection

Exploring the connection between coping songs and self-regulation goals requires assembling a large dataset of self-selected songs for achieving various well-being goals associated with mood regulation from a large number of individuals. This is challenging, as it requires obtaining information from many individuals on their coping songs, as well as information on their mood and on their self-regulation goals. Asking people to explicitly indicate what song would help them obtain a specific affective goal, could result in cognitive biases such as time-relatedness (e.g., recency, i.e., the song was heard recently), or social biases (responding according to what is socially appropriate or is expected of them). Overcoming these biases requires careful manipulations which are hardly feasible for a large-scale data collection.

Another option for obtaining such data could presumably be using the Experience Sampling Method in which participants are asked to fill-out questionnaires several times in response to experimenters’ scheduled cues. This enables to capture music listening in “natural settings” over very differing contexts and situations as indeed has been successfully implemented in a number of studies (e.g., Juslin et al., 2008; Randall et al., 2014). Yet, this method has its drawbacks, as respondents are very much aware of participating in a study and can probably easily discern its goals.

For the current paper, we used a large-scale unique dataset assembled by Granot et al. (2021), following the first COVID-19 pandemic lockdown, which monitored participants’ emotional states as well as their coping mechanisms. Granot et al. (2021) administered a 15-min online questionnaire during the first wave of the COVID-19 pandemic (between July and November 2020), to a convenience sample of 5,619 participants from 11 countries that were severely affected by the pandemic: Argentina, Brazil, China, Colombia, Italy, Mexico, the Netherlands, Norway, Spain, the UK, and the US. The questionnaire was available in six languages (Chinese, Dutch, English, Italian, Norwegian, and Spanish). As explained to respondents, the survey was intended to “gain a better understanding of how people cope with the COVID19 crisis by using daily activities, especially music, in order to reduce negative feelings and maintain wellbeing in these complex times.” The questionnaire contained seven sections that examined respondents’ feelings, emotions, and coping activities besides music, during COVID-19 lockdown.

#### Coping songs

Our focus is on the question that was not used in the Granot et al. (2021) paper, which asked participants to provide the title of/link to the song that helped them cope most during lockdown (see Supplementary Appendix A). If the name of the song was provided instead of a link, we searched for the song and used the most popular version. Of the entire sample, we removed 1,366 participants who did not provide a link to/title of a song. As our study analyzes lyrics, we retained only the 2,804 responses that included songs with lyrics. Note that the survey captured a snapshot of coping songs at a specific point in time. It did not inquire about the use of coping songs pre- pandemic, nor did it track changes in use throughout the entire lockdown.

#### Well-being goals

For each of the 2,804 participants we used the “Goals” section from Granot et al.’s (2021) questionnaire. This section, being central to the study, always appeared first, (with other five sections presented in a randomized order). Participants were asked to rank how important on a five-point Likert scale (from 0 “irrelevant” to 4 “to a very large degree”) five wellbeing goals were in their coping with the lockdown and the situation that was imposed on them. The goals were: release and venting of negative emotions (e.g., stress, anxiety, anger); Diversion from the crisis; Enjoyment and maintaining good mood; Reducing loneliness and creating a sense of togetherness; Connecting with myself and detachment from the surroundings. Participants could also add goals in a blank space but these did not reach 2% of the sample.

#### Participants’ sample statistics

The number of respondents per country who provided a song link/title ranges from 83 (China) to 498 (Colombia), with a median of 250. On average, two-thirds of the respondents were female (range between countries 50–76%); 30% were younger than 24 years old; 45% were 25–44; 20% were 45–64; and 4% were over 64. About 34% of respondents reported having a low level of spirituality, compared with 66% who reported a medium or high level. Most respondents assigned a high level of importance to music (75% between 4 and 5), while only 8% assigned it a low level (between 1 and 2). See Supplementary Appendix B for complete descriptive sample statistics.

### Extracting songs’ lyrics and genre

Lyrics were extracted using a YouTube plugin we developed for the project. A designated script first automatically opened the song’s YouTube link, and then used the plugin to extract the lyrics (which in most cases are displayed in the information section underneath the video box), into a dedicated file. Lyrics in languages other than English (953 out of 2,804), were translated into English using Google Translate.

The genre of each song was retrieved using PyTube, a Python library which obtains the video title from the YouTube link in the dataset. We then created a Python script to run ChatGPT to extract the song name and artist from each video title. As a last step, we ran a Python script to obtain from ChatGPT the genre of the song using the song name and artist.

### Lyrics analysis

Content analysis of songs’ lyrics is challenging due to their non-standard grammatical structure, the limited number of words in each song, and the repetition of words and phrases. Thus, common topic modeling approaches such as LDA (Kleedorfer et al., 2008; Sasaki et al., 2014; Fell et al., 2019, 2022; Nakai and Itoh, 2022) often fail to model the topic structure in corpuses of songs. We therefore implemented Non-Negative Matrix Factorization approach (NMF), an unsupervised topic modeling algorithm that simultaneously performs dimensionality reduction and clustering (George and Vasudevan, 2020). This model regards the song corpus as a collection of documents (M’sik and Casablanca, 2020), and classifies them into a predefined number of topics. Each topic contains all of the words in the corpus, with differing weights, arranged in descending order so that the most dominant words in each topic receive the highest weights.

As a preliminary stage, we performed a massive cleaning of the data to omit stop words (e.g., “is,” “the,” “chorus”), artists’ names, syllables that are not words (e.g., “haa,” “wawa”), punctuation marks, and numbers. We also performed a stemming procedure wherein inflected words are reduced to their roots (e.g., “likes” or “liking” became “like”). The cleaning process resulted in a lexicon of 6,000 words. For the lyric analysis, songs that were chosen by multiple respondents and repeated multiple times were removed so that each song occurs only once. This is important, so that the model is not biased toward songs that were chosen several times. Ultimately, the model was applied to 2,386 songs.

There is no established way of determining the number of topics. We tried various options between 1 and 30 topics, and calculated the coherence score for each such run. The coherence score is a measure of the probability of the topics be interpretable by humans. The higher the probability, the higher the likelihood that the topics can be interpreted (i.e., that people are able to comprehend the topic’s underlying theme) (gensim.models.CoherenceModel library). Based on the coherence graph, we set the number of topics at 15. See Supplementary Appendix C for the coherence score graph.

### Interpretation of topics themes

While topic modeling algorithms do not suggest a procedure for interpreting the resulting topics, to facilitate interpretation and communication, we named the topics using a three-stage process. First, we presented the 10 words with the highest weights in each of the 15 topics to 40 MTurk respondents (paid $1 each), and asked them to suggest a title for the topic based on the commonality between the words. We then fed the words into ChatGPT and asked it to suggest titles using the prompt: “Find a common denominator between the following 10 words: …” We integrated the responses to obtain 15 topic titles. In the third stage, we conducted an MTurk experiment wherein we presented to 210 MTurk respondents the top 10 words of the topic, and asked them to choose between the suggested topic title and an alternative title, to be chosen randomly from the titles suggested for the other topics (this design makes sense, as in topic modeling, all of the words in the lexicon are part of all the topics, but in differing weights). The overwhelming majority of the respondents chose the suggested title over its alternative (see Supplementary Figure D1). Note that, despite this procedure, topic naming is not an inherent part of topic modeling, and alternative naming approaches could result in slightly different topic names.

### Lyrics and well-being goals

We regressed the goals against the lyric topics. For each of the 15 topics *i* = {1, 2, … 15}, we ran a linear regression wherein the dependent variable had the frequency *y_ij_* of the topic *i* in the coping song chosen by respondent *j*, and the independent variables were the respondents’ ratings of each of the five goals. The control variables $\bar{X}$ include respondents’ demographics (age, gender, number of children, personal status), as well as their levels of religiosity and spirituality, “Religiosity” indicating prayer, reading religious texts, and attending worship “Spirituality level” meaning sense of meaning and purpose in life beyond material values as indicated on a 5-point scale: not at all…very strongly. We added the interaction terms $\bar{\beta }$ between the goals and the degree of music’s importance to the respondent. The complete model for topic *i* is:


$$y_{ij}=\alpha_{0}+\bar{\alpha }\;.\;{\bar{\mathrm{goal}}}_{j}+\bar{\beta }\;.\;{\bar{\mathrm{goal}}}_{j}\;.\;{\bar{\mathrm{music}}}_{j}+\bar{\gamma }\;.\;{\bar{X}}_{j}$$

As the regression results created a very specific matching of topics to goals, and due to the large number of regressions, we ran a Placebo Permutation Test (Abadie and Gardeazabal, 2003) to verify that the results indeed indicated a specific matching and were not a random outcome. Therein, the independent variables in each observation—which were the respondent’s ratings of the five goals—were randomly permutated. Thus, we maintained the respondent’s average rating level while randomly permutating her responses across the five goals. These permutated results serve as a placebo test, i.e., running the regression on these permutated data should yield zero significant coefficients, which is indeed what we obtained.

### Extracting song’s acoustic features

The literature suggests a wide range of high- and low-level acoustic features (Han et al., 2022). For better interpretability, we chose features that were found to influence the general emotional response to the song (Eerola, 2011, 2012). For example, a song with high volume, fast tempo, little roughness, simple harmony, and tendency toward the Major mode, would likely be perceived as “happy” and could be used to maintain one’s good mood. Loud, fast, heavy bass, and rough sounds in a song would likely be perceived as threatening or reflecting anger, and thus might help to vent negative emotions (Juslin et al., 2015; Karreman et al., 2017).

We used the Essentia (Bogdanov et al., 2013) audio analysis library to extract musical features, encompassing spectral, tonal, and rhythmic descriptors. Analysis was run on WAV files extracted from the YouTube links. Two types of acoustic features were extracted: Five features for a general characterization of the dataset, and another set of five features for testing the possible associations to well-being goals.

The features for the data set characterization included song-length, estimated key in which the song is written (e.g., E flat), mode (major versus minor), “danceability” (reflecting beat regularity and strength), and tempo. Danceability was measured on a scale of 0–3 with 3 being “more danceable.” We defined the tempo as “slow” for average of 80 BPM, “medium” as 80–120 BPM, and “fast” as over 120 BPM.

For the main analysis we extracted 161 low-level musical features that refer to spectral, tonal, and rhythm descriptors. As each feature has a differing range of values, we normalized the values to the range between 0 and 1. For our purposes, we chose to focus on 5 main feature groups: Loudness - the volume of the song; Mode - major/minor; Tempo - based on the beat duration (fast/slow); Harmony - the degree of harmony in the song; and Timbre—bright/dark sound. For each parameter, we calculated the average of their values. See Supplementary Appendix E for the exact features used for each parameter.

### Acoustic features and well-being goals

For each of the 5 feature groups *i* = {1, …, 5}, we ran a linear regression wherein the dependent variable was the average value *y_ij_* of the feature group *i* in the coping song chosen by respondent *j*; and the independent variables were the respondent’s rating of each of the five goals. The control variables included respondents’ demographics (age, gender, number of children, personal status), as well as their levels of religiosity and spirituality. The regression model for each feature group was:


$$y_{ij}=\alpha_{0}+\bar{\alpha }\;.\;{\bar{\mathrm{goal}}}_{j}+\bar{\gamma }\;.\;{\bar{X}}_{j}$$

## Results

In the first part of the results, we provide descriptive information about the coping songs in terms of genre, artists, and acoustic features. We then present the 15 topics of the coping songs’ lyrics, emerging from the NMF topic modeling. Finally, we present results for the main question in which we were interested: the relationship between participants’ well-being goals and the lyrics and acoustic features of their nominated coping songs.

### Coping songs’ characteristics

**Figure 1:**
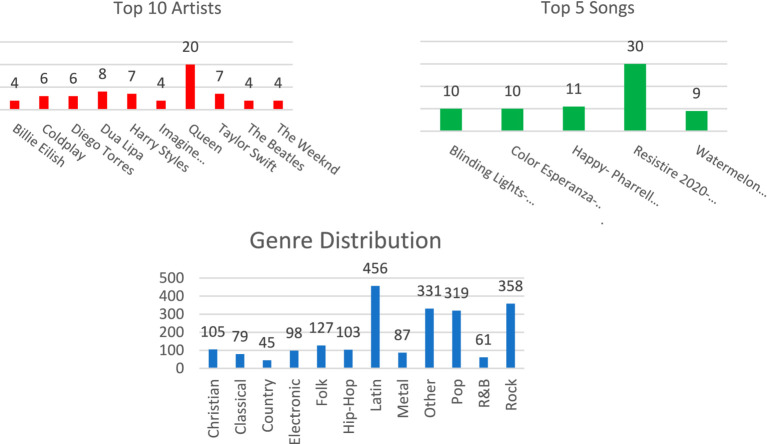
The top five songs, top 10 artists, and genre distribution of the nominated coping songs.

**Figure 2:**
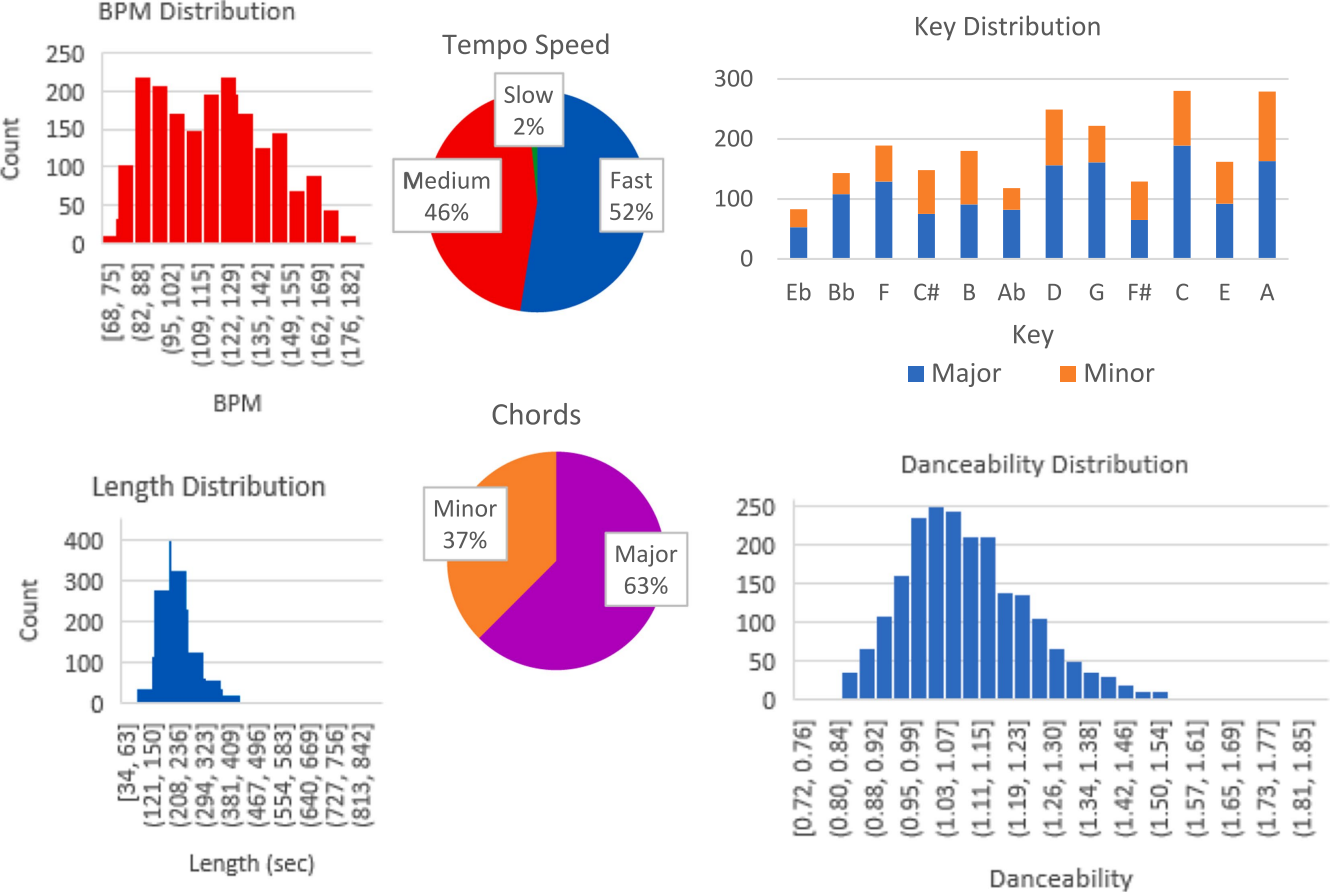
The distribution acoustic features of the nominated coping songs. The figure indicates that the coping songs varied greatly in their musical features, mostly dominated by a major key, medium-to-fast tempo, and low danceability.

As shown in Figures 1 and 2, the distribution of coping songs spans a wide range of artists and musical genres. The popular genres were Latin, Pop, and Rock, where the strong presence of Latin might be attributed to the high representation of Spanish-speaking respondents.

### Lyrics analysis: 15 emerging topics

We used the model on the extracted lyrics of the coping songs to identify 15 emerging topics that reflect the self-organization of the lyrics in the song corpus. Table 1 shows the 10 words with the highest weights in each topic. While extracted topics from topic modeling algorithms are not easily interpreted, and for many purposes do not require naming, herein, for ease of communication and better interpretability, we applied a 3-stage procedure using human encoders to identify the topic (see Methods for details on the identification procedure and validation experiments). The 15 topics named are: Soul, Time, Desire, Thought, Loneliness, Breakup, Love Story, Absolute, Ups and Downs, Optimism, Friendship, Life and Death, Belonging, Loss, and Night.

**Table 1:** The 10 words with the highest weights of each topic.

Topic	1st	2nd	3rd	4th	5th	6th	7th	8th	9th	10th
Soul	Make	Way	Well	Little	Good	Take	Word	Face	Soul	Die
	0.386	0.053	0.041	0.04	0.034	0.03	0.03	0.029	0.025	0.024
Time	Time	Take	Ill	Away	Wait	Mind	Back	Good	Lose	End
	0.405	0.243	0.189	0.12	0.065	0.063	0.054	0.053	0.051	0.047
Desire	Want	See	Live	Always	Good	Stop	Take	Girl	Look	Stay
	0.767	0.056	0.044	0.03	0.03	0.028	0.028	0.025	0.025	0.023
Thought	Say	Man	Word	Think	Head	Way	Call	Around	Try	Believe
	0.937	0.107	0.099	0.09	0.076	0.071	0.069	0.066	0.061	0.06
Loneliness	Need	Keep	Wait	Tell	Mind	Right	Alone	Little	Friend	Run
	0.843	0.078	0.077	0.08	0.067	0.06	0.06	0.056	0.047	0.036
Breakup	Love	Heart	Fall	Much	Leave	Cry	Song	Dream	Last	Soul
	1.593	0.13	0.071	0.05	0.043	0.043	0.031	0.03	0.028	0.027
Love Story	Baby	Girl	Well	Keep	Tell	Man	Could	Ill	Call	Little
	0.903	0.306	0.15	0.14	0.107	0.095	0.091	0.08	0.079	0.063
Absolute	Never	World	Always	Could	Nothing	Everything	Ever	Away	Change	Lose
	1.029	0.236	0.186	0.18	0.176	0.176	0.164	0.152	0.149	0.135
Ups and Downs	Day	New	Think	Die	Man	Lose	Alone	Walk	Sun	Night
	0.936	0.102	0.075	0.06	0.052	0.049	0.048	0.047	0.045	0.041
Optimism	Fell	Heart	Good	Fall	Inside	Stop	World	People	New	Hold
	1.069	0.122	0.106	0.09	0.089	0.08	0.074	0.065	0.063	0.052
Friendship	Know	Tell	Think	Try	Look	Well	Could	Alone	Stay	Friend
	0.966	0.162	0.116	0.1	0.096	0.087	0.076	0.068	0.068	0.063
Life and Death	Life	Live	Always	Little	Everything	Sing	Die	God	People	Nothing
	1.004	0.369	0.137	0.11	0.104	0.095	0.086	0.078	0.068	0.066
Belonging	Come	Back	Home	God	Sun	Nothing	Walk	Keep	Left	Tell
	1.196	0.307	0.144	0.12	0.087	0.074	0.063	0.061	0.049	0.048
Loss	Give	Heart	Everything	Lose	God	Song	Leave	Put	Hand	Something
	1.548	0.24	0.146	0.11	0.08	0.079	0.07	0.067	0.054	0.053
Night	Night	See	Light	Eye	Look	Sky	Dream	Star	Heart	Sun
	0.581	0.575	0.485	0.48	0.432	0.378	0.338	0.307	0.253	0.25

#### Topic distribution

**Figure 3:**
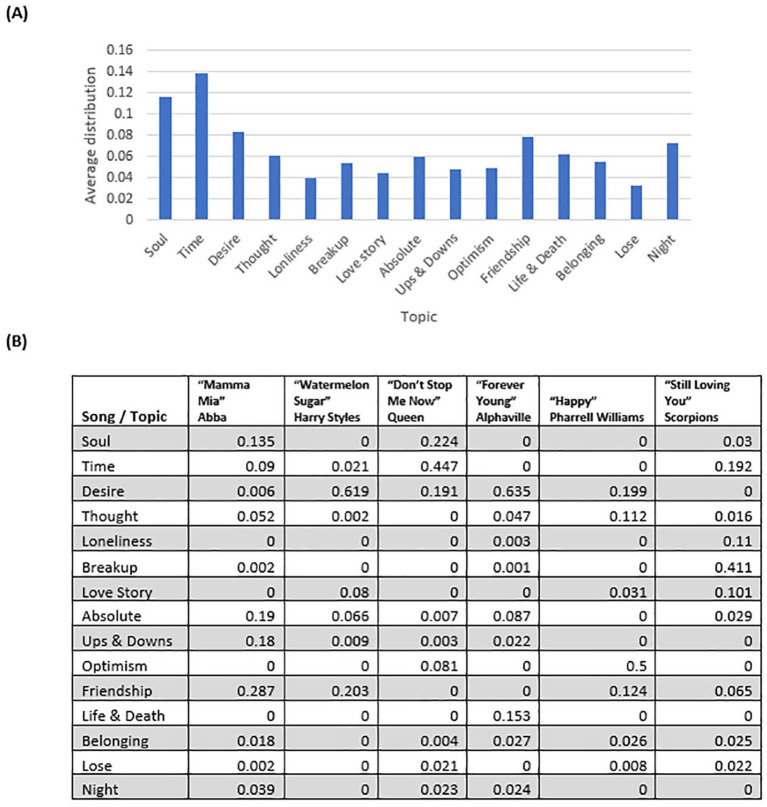
Topic distribution across all songs **(A)**, and examples of the distribution of topics within a song **(B)**.

Figure 3 presents the average distribution of topics across all songs (top). The most common topics are Soul and Time (songs such as “Natural” by *Imagine Dragons*; “No Rain” by *Blind Melon*; “Time After Time” by *Cyndi Lauper*). The bottom panel shows several examples of the distribution of the topics within a song (i.e., the normalized frequency of the topics in a song). The higher the value for a particular topic, the more dominant the topic is within that song. The song “Forever Young,” for example, is distributed 63% over the topic “Desire,” 15% over the topic Life and Death, and at far lower probabilities for the other topics (Figure 3B).

The topic distribution for specific demographic segments and countries is presented in Supplementary Appendix D. Time and Soul are the two most dominant topics for all countries, together with Desire, Friendship, and Night. Countries differ in the ubiquity of the topic Life and Death. While it was relatively prevalent in Brazil, Colombia, and Spain, its occurrence was lower in other countries (Supplementary Figure D2). For age groups, while Time and Soul were common for most age groups, for the 64+ age group, other topics such as Friendship, Night, and Life and Death were more dominant than Soul (Supplementary Figure D3). Interestingly, the topic Belonging was dominant among participants with high level of religiosity (scoring 5 on the 1–5 scale) versus those who reported/described themselves as less religious (Supplementary Figure D4). No significant differences were found in the topic distribution across genders (Supplementary Figure D5).

### Lyrics and well-being goals

The relationships between the well-being goals and the nominated coping songs were assessed using a set of 15 linear regressions over all respondents, where the dependent variable *y_ij_* is the frequency of topic *i* in the song chosen by respondent *j*, and the independent variables are the ratings of the five goals reported by respondent *j* (see Methods for the formal model). Table 2 shows the significant coefficients ( $P_{\mathrm{val}}<0.05)$ in this 15 × 5 matrix, where the positive coefficients are highlighted in green and the negative in red, with the color intensity representing the intensity of the effect. The complete table (15 regressions with 5 goals and additional control variables) is presented in Supplementary Appendix D.

**Table 2:** The significant regression coefficients of the goal variables.

		Topics							
		Absolute	Ups and Downs	Optimism	Life and Death	Belonging	Loss	Night	
Goals	Release and venting of negative emotions								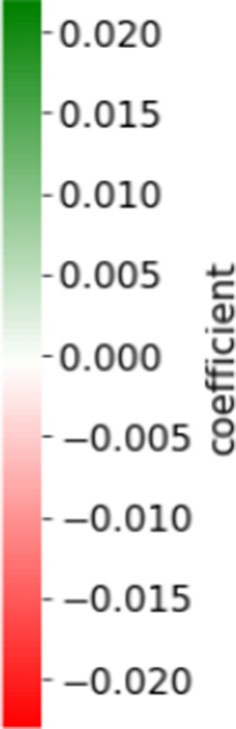
	Diversion from the crisis		−0.018				0.011		
	Enjoyment and maintaining good mood				−0.023			−0.017	
	Reducing loneliness and creating a sense of “togetherness”	0.022		−0.015					
	Connecting with myself and detachment from the surroundings					0.013			

Table 2 reveals very specific matching between lyric topics and goals. For example, the goal Diversion (from the crisis) was found to be positively associated with the topic Loss, and negatively with Ups and Downs. Hence, the higher the respondent rated her goal as Diversion from the crisis, the higher the frequency of the topic Loss, and the lower the frequency of the topic Up and Down in the coping song s/he chose. The well-being goal of Enjoyment and maintaining good mood is negatively correlated with Life and Death, and Night. The goal of Reducing loneliness and creating a sense of togetherness is positively associated with Absolute (featuring words such as “always,” “never,” “everything,” and “nothing”), and negatively with Optimism. Finally, the goal of Connecting with myself and detachment from the surroundings is positively associated with the topic Belonging. While the interpretation of these relationships depends upon the interpretation of the topics (see Discussion), the core of this finding is this very specific match between song lyrics and specific well-being goals. To further validate that these relationships are not spurious, we conducted several tests, described in more detail in the Methods section.

### Interaction with the respondent’s indicated importance of music

A possible moderator of influence that we tested was the perceived importance of music to the respondent. Granot et al. (2021) found that music’s importance in one’s life is strongly connected to the music < > well-being goals relationship, i.e., the variable of music’s importance was the strongest predictor of music’s rated effectiveness in reaching the respondent’s well-being goals, above and beyond musical education, the musical genre, and demographic variables.

Table 3 shows the significant coefficients obtained from the regressions for the interaction terms between the goals and the respondents’ response as to the music’s importance to them (“How important to you is music in general?”) (see Methods; the complete result table is in Supplementary Appendix F). Most of the significant coefficients are opposite in sign to the main effect in Table 2, indicating that music’s importance mostly moderates the goals < > topic relationships. A possible interpretation thereof is that music lovers (e.g., those who indicate that music is highly important to them), relate to the song as a whole, attributing higher relative importance to the acoustic features relative to the lyrics.

**Table 3:** The significant regression results of the interaction variables, where the number denotes the coefficient value.

		Topics							
		Absolute	Ups and Downs	Optimism	Life and Death	Belonging	Loss	Night	
Goals	Release and venting of negative emotions								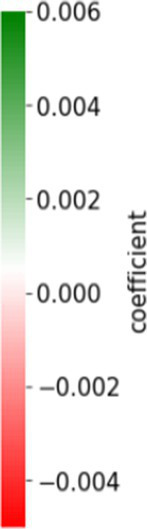
	Diversion from the crisis		0.004				−0.003		
	Enjoyment and maintaining good mood				0.006				
	Reducing loneliness and creating a sense of “togetherness”	−0.005		0.004					
	Connecting with myself and detachment from the surroundings								

The numbers denote the regression coefficient value.

### Acoustic features and well-being goals

Similar to the lyrics, we ran a set of five linear regressions (for each musical features group), where the dependent variable *y_ij_* is the frequency of group *i* in the song chosen by respondent *j*, and the independent variables are the ratings of the five goals reported by the respondent *j*, and additional control variables (see Methods for the formal model; see Supplementary Appendix E for the list of Essentia features used).

The acoustic features of music have been shown to have a strong effect on emotions (e.g., Schubert, 2004; Juslin and Lindström, 2010). Therefore, we could expect the coping song’s acoustic features to play a dominant role in achieving the well-being goals, in addition to, or even instead of, the lyrics. To test this, we assessed the connection between the respondents’ well-being goals and acoustic features of their nominated coping songs. Surprisingly, we did not find consistent relationships between well-being goals and coping songs’ acoustic features. The regression coefficients (described in Supplementary Appendix G), are mostly insignificant and do not exhibit any systematic pattern.

Note that this result does not imply that acoustic features are unimportant for well-being goals; it only means that on average, no clear pattern emerges across individuals. It could be that people differ greatly in their musical tastes and preferences, resulting in diverse acoustic features of the nominated coping songs, which blur the average choice. Moreover, the fact that the coping songs consist of many musical genres from various decades, decreases the probability of finding consistent acoustic patterns, especially for the dimension of valence (Eerola and Vuoskoski, 2012). Lyrics, on the other hand, are more explicit, i.e., the set of words that various people choose to describe loneliness is narrower than the spectrum of acoustic features that they might choose to resonate their lonely feelings. This narrower lyric choice led to the more consistent pattern in the lyrics vs. the acoustic features.

## Discussion

In this paper, we explored the relationship between people’s choice of coping songs and their stated well-being goals. We used the unique circumstances of the first wave of the COVID-19 pandemic to assemble and analyze a large-scale, multinational dataset of 2,804 coping songs nominated by 5,619 individuals from 11 countries. We implemented a topic-modeling approach to identify 15 topics that span the songs’ lyrics space. These topics were not pre-defined, but rather self-emerged from the data. For interpretability, we termed them: Soul, Time, Desire, Thought, Loneliness, Breakup, Love Story, Absolute, Ups and Downs, Optimism, Friendship, Life and Death, Belonging, Loss, and Night. We found specific matching between respondents’ well-being goals and the incidence of the related topics in the lyrics of their nominated songs (Table 2).

Is there a unifying overarching pattern to the results in Table 2? While this paper does not aim to test specific hypotheses, two possible explanations come to mind. The first suggests that both the well-being goals and the coping songs were driven by a third factor related to the individual’s emotional state. This emotional state induces both the well-being goal, as well as the nominated coping song. For example, an individual who feels overwhelmed by the crisis might tend more to choose a song with the topic Loss, and at the same time might also feel the need to set a goal of Diversion from the crisis. When happy, one will set the goal of Enjoyment and maintaining good mood, and in turn, choose songs less related to Life and Death. Thus, the well-being goals and coping songs are connected via the listener’s underlying emotional state. This explanation is consistent with recent findings showing that lyrics of favorite songs are associated with personality traits and attachment style (thoughts, feelings, and behavior related to close relationships; Alaei et al., 2022), and hence may help to fill psychological needs (Qiu et al., 2019).

An alternative explanation suggests that well-being goals are regarded as a lever for moving from one emotional state to another. To help in this transition, and specifically, where the existing emotional state is already negative, people select a coping song that reflects their *existing* emotional state rather than their *desired* one. They can then engage in any of the emotion-focused strategies ranging from expression and ventilation, to reappraisal, acceptance, or even suppression (Gross, 2015). However, they do this within the safe environment of the songs rather than while confronting the real-life stressful situation. This is consistent with Scheff’s (1979) view of Catharsis that contains three stages: The first, evocation of the negative emotions through music (or drama, as in Aristotle); the second is the distancing of the listener as separate from the virtual protagonist, enabling cognitive 3rocessing that is at the heart of the therapeutic stage; and the third is the emotional release that may include crying, laughing, or other somatic reactions.

Our results support the latter explanation. For example, someone who seeks Diversion from the crisis likely feels immersed in/overwhelmed by the crisis. As per our findings, such an individual will tend to choose a negative valence topic such as Loss, which reflects the crisis, and is less likely to choose the topic of Ups and Downs, which may indicate an attempt to achieve a more optimistic outlook. Similarly, someone who seeks to Reduce Loneliness and Create a Sense of Togetherness, feels lonely, and thus will be less inclined to choose lyrics with Optimism as a dominant topic, and more inclined toward the topic Absolute, which includes existential words such as “never” or “nothing,” that actually reflect her existing emotional state. An individual whose goal is Connecting with myself and detachment from the surroundings might have chosen this goal due to feeling engulfed by those around her, while the topic Belonging, which conveys the theme of attachment to home or a community, reflects the situation of being surrounded by others.

For the goal Enjoyment and maintaining good mood, the existing and desired states are similar, and the song selection reflects both. Someone seeking to maintain her good mood will tend less to select lyrics with topics such as Life and Death and Night that might place her farther from a good mood.

These findings are consistent with Thoma et al. (2012) who found that given a choice, listeners prefer mood-congruent musical pieces, which can lead to positive results through providing a sense of meaning for the negative emotion, e.g., feeling less lonely (“I’m not the only one experiencing these feelings”), acceptance, and deeper processing of the emotion (Baltazar and Saarikallio, 2019; Garrido et al., 2022). More generally, according to the instrumental approach to emotion regulation, individuals may choose to experience negative emotions if they expect this to help them obtain a desired goal such as feeling anger in preparation for confrontation, or worry if they believe that avoidance is beneficial (e.g., Tamir and Ford, 2012).

Note that both these explanations depend upon the interpretation and naming of the topics. While such naming has been done in other contexts (Dzyabura and Peres, 2021), one should be cautious when relying on these interpretations so as to infer psychological mechanisms. The power of our findings is the *specific* matching between goals and lyrics topics. It is not necessary that all of the matchings be a result of the same coping strategy, and it could well be that individuals employ differing coping song strategies for differing well-being goals.

While the goal < > lyrics relationships show a clear, specific matching, we did not find clear matching between the well-being goals and the coping songs’ acoustic features. Accordingly, Table 3 indicates that the effect size is smaller for those who rated music as important in their lives. For those individuals, focusing on the musical elements distracts them from lyrics, thus weakening the lyrics < > goals relationship. It seems that while lyrics have a clearer/more consistent interpretation across listeners, music is by nature abstract, lending itself to a broad range of interpretations. Thus, while one individual might use an energetic tune to reach the goal of Diversion from the crisis, another might choose a song featuring a more serene musical ambience to achieve the same goal. This heterogeneity, however, does not converge into a unified average pattern of musical choice.

## Limitations and future research

This study utilized a unique, large-scale ecological dataset of coping songs, employing a bottom-up thematic and acoustic analysis focused on emergent self-patterns. However, this approach has certain limitations. First, as an exploratory study, the scope of mood regulation goals was relatively limited. Future research could broaden these goals and incorporate questionnaires that assess individual differences in emotion regulation strategies and empathy. Additionally, our analysis was based on a single time point. Examining the use of the same coping song over time would provide insights into its dynamic role in mood regulation.

As noted in the Methods section, we limited our analysis to a select number of musical features. Including a more extensive set of features and employing more sophisticated analytical methods might reveal more stable patterns. The greatest challenge—and possibly the most ambitious—is finding a way to analyze music and lyrics as a unified expression, as it is likely the unique interaction between them that drives their impact.

Regarding lyrics analysis, we acknowledged the complexity in defining thematic topics meaningfully, a challenge further intensified by the need for translations in approximately one-third of the songs. Nonetheless, a separate analysis of non-translated lyrics showed consistent patterns. Future studies should continue investigating the role of lyrics in music listening and music therapy contexts.

## Conclusion

In stressful times, people often find solace in coping songs, or songs that they listen to repeatedly to help them face hardship. Our data-driven lyrics analysis indicates that coping songs’ lyrics relate to specific topics, which we name as Soul, Time, Desire, Thought, Loneliness, Breakup, Love Story, Absolute, Ups and Downs, Optimism, Friendship, Life and Death, Belonging, Loss, and Night. These topics vary across cultures, ages, and religiosity. We found a significant association between certain topics and specific well-being goals. We demonstrate this for a large-scale dataset of 2,804 songs from various genres and eras, in multiples languages, collected from a large variety of individuals in 11 countries in the real-life circumstances of the first wave of the COVID-19 pandemic.

Our findings open a window onto a novel perspective on music classification, very different from the traditional acoustic genre-based categorization. We suggest that for emotional well-being purposes, classification based on *lyrics topics* might be valuable. Such classification could improve the matching between songs and affected individuals, and could enhance the effect of music through lyrics-driven pairing in music apps, playlist creation, and musical events.

## Data Availability

The data analyzed in this study is available at: https://osf.io/c6zge/?view_only=5ee154c047f743a8a779a601dc8c733e.

## Footnotes

^1^For example, an analysis of Spotify’s snapshot data of 32,833 popular tracks from 2020 indicates that the average instrumentalness (that is, the probability that a Spotify track does not contain vocals) is 0.084, i.e., the overwhelming majority of the popular Spotify tracks contain lyrics, see https://rstudio-pubs-static.s3.amazonaws.com/831415_8e26d20044e343efb68b42048a136bf6.html
