# Supplementary material for: Lyrics do matter: how “coping songs” relate to well-being goals. The COVID pandemic case
Source: Front Psychol. 2024 Dec 23;15:1431741. doi: 10.3389/fpsyg.2024.1431741 (PMC11701978; doi:10.3389/fpsyg.2024.1431741)
Supplement: Supplementary file 1 [file Data_Sheet_1.pdf]

Lyrics Do Matter: How “Coping Songs” Relate to Well-Being Goals. The COVID Pandemic Case

Adi Levy, Roni Granot, and Renana Peres

Supplementary Information

Appendix A

Table A1: The relevant questions from the questionnaire: Ranking the relevance of 5 well-being goals to their coping with the lockdown, ranking the importance of music, and nominating the coping song

Q20 **Achieving well-being related goals during the lockdown (3-4 min)** Please indicate the degree to which the following goals were relevant to your coping with the lockdown.

	Irrelevant 0	A small degree 1	Some degree 2	Large degree 3	Very large degree 4
Release and venting of negative emotions (e.g. stress, anxiety, anger)	○	○	○	○	○
Diversion from the crisis	○	○	○	○	○
Enjoyment and maintaining good mood	○	○	○	○	○
Reducing loneliness and creating a sense of "togetherness"	○	○	○	○	○
Connecting with myself and detachment from the surroundings	○	○	○	○	○
Other (please write down in the blank space)	○	○	○	○	○

Q95

Using music during the crisis (3-4 min)

How much is music important to you in general?

- ○ Not at all important
- ○ Slightly important
- ○ Moderately important
- ○ Very important
- ○ Extremely important

Q117 Please write down a song/piece that helped you cope most during the lockdown. YouTube link or name of song/piece (preferable a link)

____

Appendix B

Sample descriptive statistics

Variable	*Mean(%)*	*std.Dev*	*Min*	*Max*
**Release and venting of negative emotions**	3.52	1.19	1	5
**Diversion from the crisis**	3.13	1.2	1	5
**Enjoyment and maintaining good mood**	3.96	0.95	1	5
**Reducing loneliness and creating a sense of togetherness**	3.38	1.19	1	5
**Connecting with myself and detachment from the surroundings**	3.31	1.17	1	5
Gender (male)	33%	-	-	-
Gender (female)	65.5%	-	-	-
Gender (prefer not to say)	0.5%	-	-	-
Gender (prefer to self-describe)	1%	-	-	-
Age (Less than 24)	30%	-	-	-
Age (25-44)	45.5%	-	-	-
Age (45-64)	20%	-	-	-
Age (over 64)	4.5%	-	-	-
Personal status (unpartnered)	42%	-	-	-
Personal status (in a relationship)	20%	-	-	-
Marital status (married or civil partnership)	30%	-	-	-
Personal status (divorced)	4%	-	-	-
Personal status (widowed)	3%	-	-	-
Personal status (other)	1%	-	-	-
Children (0)	65%	-	-	-
Children (1)	16%	-	-	-
Children (2)	12%	-	-	-
Children (3)	5%	-	-	-
Children (4+)	2%	-	-	-
**Religiosity *level***	2.11	1.21	1	5
Spirituality level	3.12	1.29	1	5
Music importance	4.08	0.97	1	5

Appendix C: Topic modeling coherence scores

The graph presents the coherence score as a function of the number of topics. While the highest point in the graph is 5 topics, this number does not provide enough granularity, and therefore we chose the next peak, which was 15 topics.

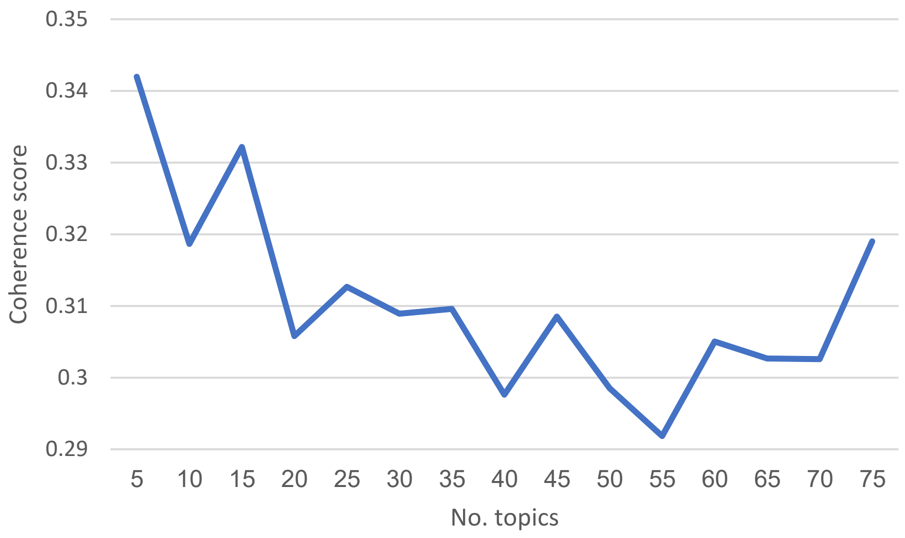

Appendix D

Figure D1. Topic name validation survey results – ratio of respondents who chose the suggested topic name over the alternative

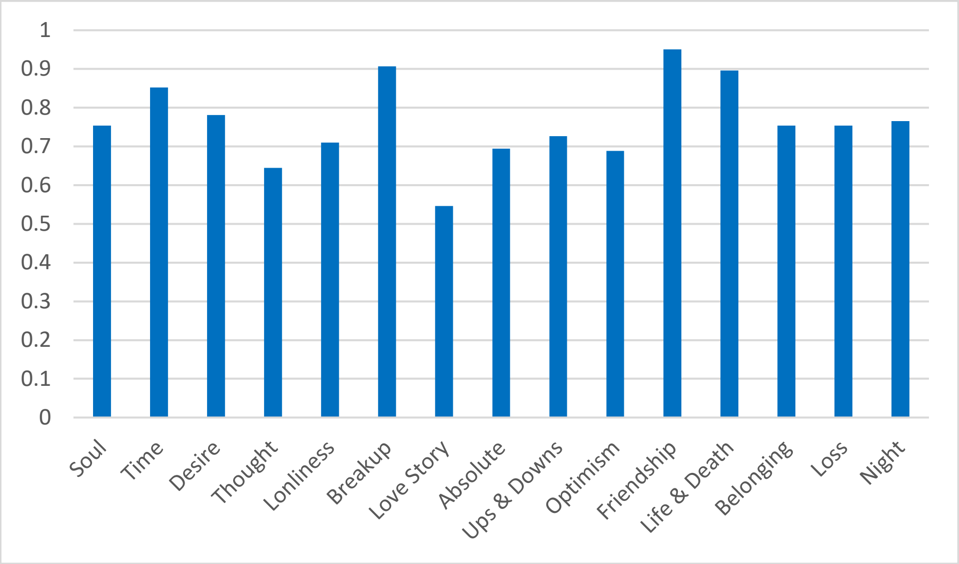

Figure D2. Distribution of topics across countries

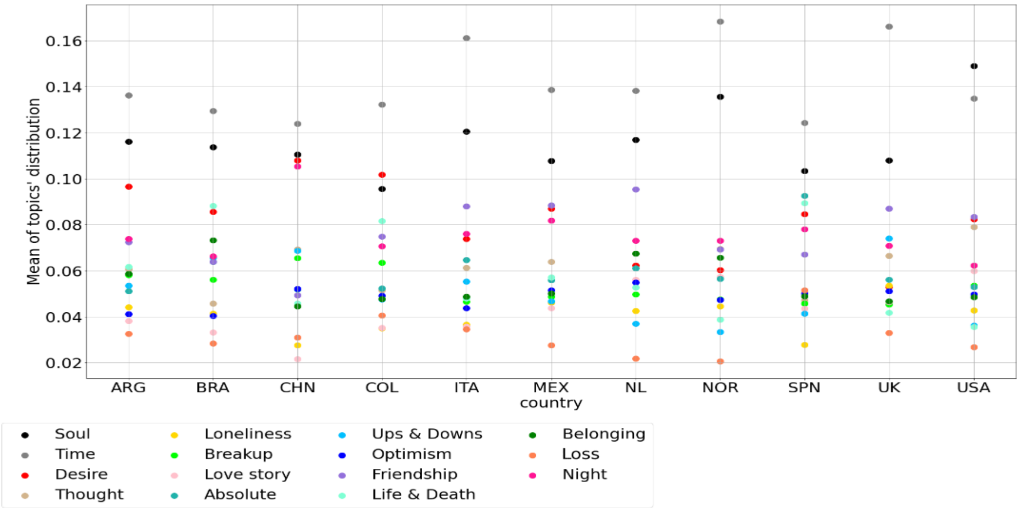

Figure D3. Distribution of topics across age groups

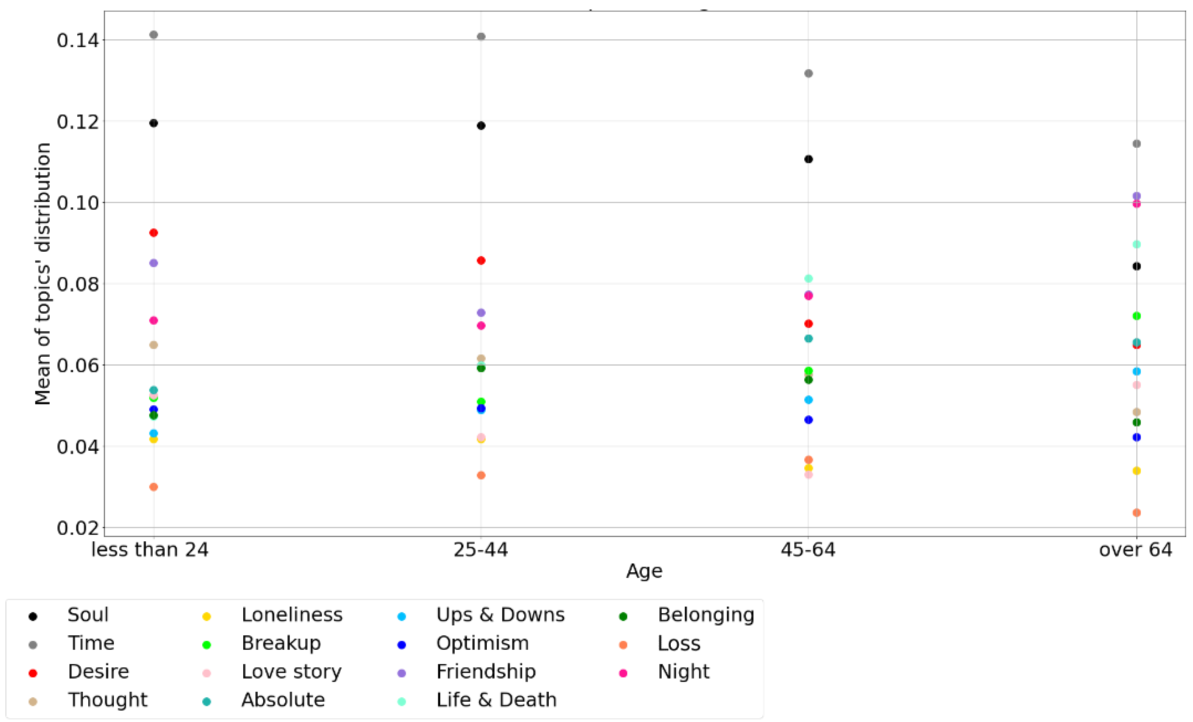

Figure D4. Distribution of topics across reported religiosity level

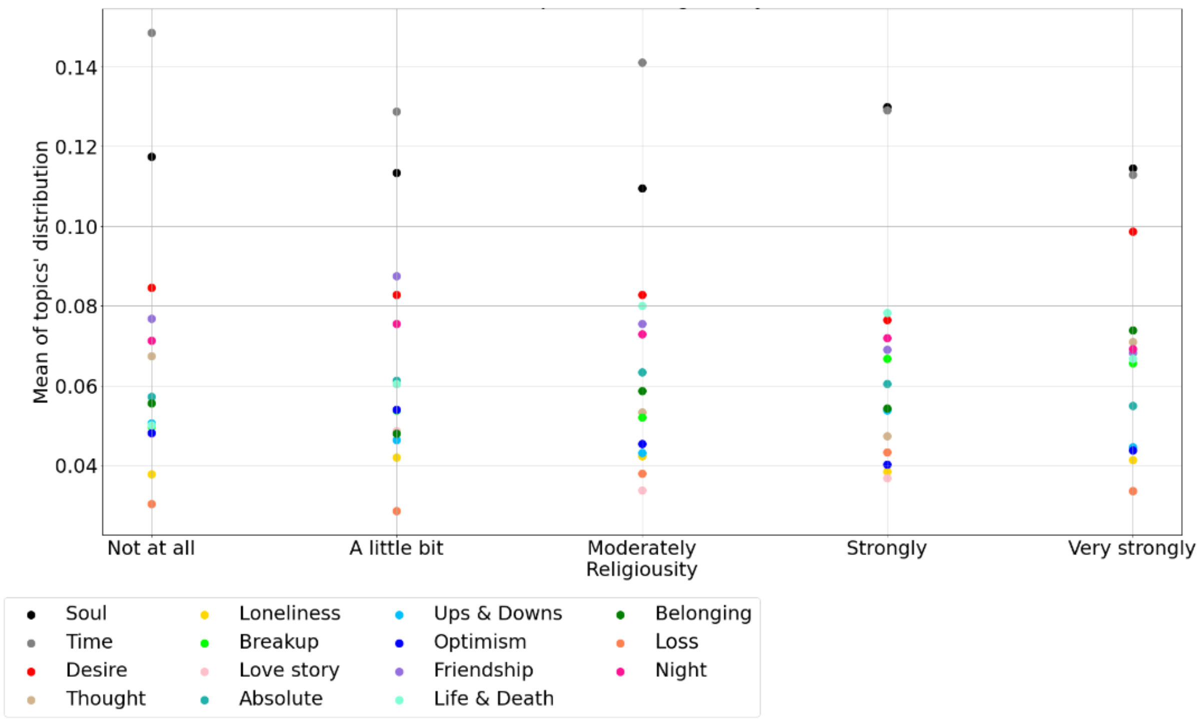

Figure D5. *Distribution of topics across reported gender*

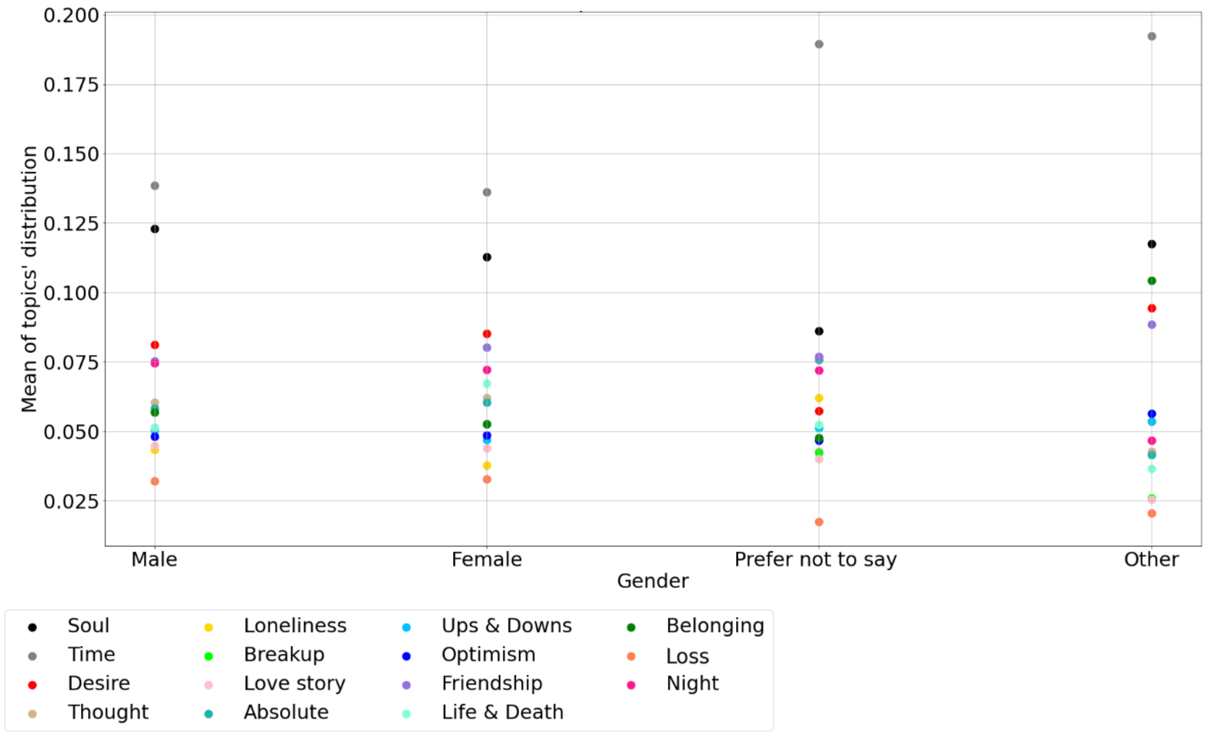

Appendix E

The Essentia features used in each group

Group name	Essentia features
Volume	lowlevel.average_loudness
	lowlevel.loudness_ebu128.integrated
	lowlevel.spectral_rms.mean
Mode	tonal.chords_scale
Tempo	rhythm.bpm
	rhythm.danceability
	rhythm.onset_rate
Harmony	tonal.chords_changes_rate_new
	tonal.chords_strength.mean
	tonal.key_edma.strength
	tonal.key_krumhansl.strength
	tonal.key_temperley.strength
Timbre	lowlevel.dissonance.mean_new
	lowlevel.spectral_centroid.mean
	lowlevel.spectral_energyband_high.mean
	lowlevel.spectral_energyband_low.mean_new
	lowlevel.zerocrossingrate.mean_new

Appendix F: Results of 15 regressions (one for each topic): Topic frequency as a function of well-being goals (N=2,804) *p<0.1; **p<0.05; ***<p<0.01

	Soul		Time		Desire		Thought		Loneliness		Breakup		Love story		Absolute		Ups & Downs		Optimism		Friendship		Life & Death		Belonging		Loss		Night	
**Independent variables**	B	SE	B	SE	B	SE	B	SE	B	SE	B	SE	B	SE	B	SE	B	SE	B	SE	B	SE	B	SE	B	SE	B	SE	B	SE
Intercept	.125***	.017	.143***	.018	.086***	.015	.093***	.011	.039***	.010	.047***	.011	.072***	.010	.039***	.010	.047***	.011	.053***	.010	.083***	.012	.027*	.012	0.033**	.011	.021*	.008	.088***	.012
Release and venting of negative emotions	-.004	.012	.007	.012	-.013	.010	-.001	.008	-.003	.007	.001	.008	.001	.007	.003	.007	.004	.008	.004	.007	-.011	.009	.009	.008	.001	.008	-.007	.006	.010	.008
Diversion from the crisis	-.002	.012	-.010	.012	.006	.010	.007	.007	-.008	.006	.004	.007	-.001	.007	-.003	.006	-0.018*	.007	.004	.007	.008	.008	.003	.008	-.008	.007	.011*	.005	.001	.008
Enjoyment and maintaining good mood	.016	.012	.005	.012	.010	.010	.005	.008	-.002	.007	-.002	.008	.008	.007	-.009	.007	.005	.008	.010	.007	-.006	.009	-.023**	.008	-.011	.008	-.008	.006	-.017*	.008
Reducing loneliness and creating a sense of "togetherness"	-.003	.012	-.007	.012	-.001	.010	-.010	.008	.012	.007	-.001	.008	-.005	.007	.022**	.007	-.003	.008	-0.015*	.007	.000	.009	.006	.008	.011	.008	.010	.006	-.004	.008
Connecting with myself and detachment from the surroundings	-.007	.011	.003	.011	.003	.009	-.005	.007	-.004	.006	.001	.007	-.006	.006	-.005	.006	.006	.007	-.002	.006	.010	.008	.004	.007	.013	.007	-.001	.005	.004	.007
Release and venting of negative emotions * music_importance	.001	.003	-.002	.003	.003	.002	.000	.002	.000	.002	.000	.002	-.001	.002	.000	.002	-.001	.002	-.001	.002	.003	.002	-.001	.002	.000	.002	.002	.001	-.002	.002
Diversion from the crisis * music_importance	.001	.003	.003	.003	-.002	.002	-.001	.002	.002	.002	-.002	.002	.000	.002	.001	.002	.004*	.002	-.001	.002	-.001	.002	-.002	.002	.002	.002	-0.003*	.001	-.001	.002
Enjoyment and maintaining good mood * music_importance	-.004	.003	-.001	.003	-.001	.002	-.002	.002	.000	.002	.001	.002	-.002	.002	.002	.002	-.002	.002	-.003	.002	.001	.002	.006**	.002	.003	.002	.002	.001	.003	.002
Reducing loneliness and creating a sense of "togetherness" * music_importance	.001	.003	.002	.003	-.001	.002	.002	.002	-.003	.002	.001	.002	.001	.002	-.005**	.002	.001	.002	.004*	.002	-.001	.002	-.001	.002	-.003	.002	-.002	.001	.001	.002
Connecting with myself and detachment from the surroundings* music_importance	.002	.003	.000	.003	-.001	.002	.001	.002	.001	.001	.000	.002	.002	.001	.001	.001	-.002	.002	.001	.001	-.002	.002	-.002	.002	-.003	.002	.000	.001	-.002	.002
Age (25-44)	-.007	.008	.006	.008	-.003	.007	-.004	.005	.002	.004	-.004	.005	-.009*	.005	.005	.004	.004	.005	-.002	.004	-0.011*	.006	.009	.005	0.012*	.005	.000	.004	.002	.005
Age (45-64)	-.019	.011	.002	.011	-0.02*	.009	-.007	.007	-.005	.006	.002	.007	-.018**	.006	.013*	.006	.004	.007	-.006	.006	-.006	.008	.024**	.008	.010	.007	.001	.005	.013	.007

	Soul		Time		Desire		Thought		Loneliness		Breakup		Love story		Absolute		Ups & Downs		Optimism		Friendship		Life & Death		Belonging		Loss		Night	
Age (Over 64)	-.048**	.018	-.018	.018	-.027	.015	-.016	.011	-.003	.010	.015	.011	.006	.010	.013	.010	.012	.011	-.011	.010	.020	.012	.03*	.012	.000	.011	-.014	.008	.036**	.012
Gender (female)	-.012	.007	-.002	.007	.006	.006	.004	.004	-.006	.004	-.005	.004	.001	.004	.001	.004	-.004	.004	.000	.004	.006	.005	.014**	.005	-.005	.004	-.001	.003	-.002	.004
Gender (not to say)	-.044	.032	.049	.033	-.029	.028	-.021	.020	.018	.018	-.012	.021	-.010	.019	.020	.018	.003	.020	-.003	.018	-.001	.023	.011	.022	-.006	.020	-.015	.015	.003	.021
Gender (self describe)	-.008	.031	.053	.031	.013	.026	-.016	.019	.011	.017	-.032	.020	-.023	.018	-.014	.017	.003	.020	.006	.018	.012	.022	-.010	.021	.051**	.019	-.013	.015	-.029	.021
Personal status (in a relationship)	.012	.008	.005	.008	-.008	.007	-.004	.005	.002	.005	-.003	.005	-.002	.005	.003	.005	-.005	.005	.001	.005	-.004	.006	.008	.006	-.002	.005	.006	.004	-.002	.006
Personal status (married/civil partnership)	.006	.010	-.007	.010	-.010	.008	.007	.006	-.006	.006	.002	.006	.005	.006	.007	.006	-.001	.006	.007	.006	.000	.007	.003	.007	.001	.006	-.001	.005	-.007	.007
Personal status (divorced)	.031	.017	-.003	.018	-.016	.015	-.013	.011	.002	.010	-.002	.011	.003	.010	.001	.010	-.007	.011	.004	.010	-.022	.012	.015	.012	.011	.011	.005	.008	-.006	.012
Personal status (widowed)	.008	.018	.036*	.018	-.017	.015	.007	.011	-.013	.010	.002	.011	-.006	.010	.007	.010	-.002	.011	-.007	.010	.007	.013	-.010	.012	-.001	.011	.004	.008	-.005	.012
Personal status (other)	-.002	.027	-.013	.027	.019	.023	.017	.017	-.036*	.015	-.010	.017	.022	.016	.001	.015	.009	.017	.012	.015	.016	.019	-.026	.019	-.006	.017	-.001	.013	-.017	.018
Children (0)	.011	.011	-.003	.011	-.012	.009	.003	.007	-.001	.006	.001	.007	-.002	.006	-.003	.006	.004	.007	.002	.006	-.002	.008	.008	.007	-.004	.007	.000	.005	-.003	.007
Children (1)	.001	.011	-.004	.011	.006	.009	-.004	.007	.006	.006	-.001	.007	-.003	.006	-.007	.006	.010	.007	-.001	.006	.012	.008	-.002	.008	-.003	.007	.003	.005	-.004	.007
Children (2)	.001	.016	-.003	.016	.014	.014	-.013	.010	.003	.009	.012	.010	-.003	.009	-.013	.009	.001	.010	.004	.009	-.019	.011	.018	.011	-.012	.010	.015	.008	.006	.011
Children (3)	.025	.023	-.003	.023	.009	.020	.009	.014	-.016	.013	-.017	.015	-.019	.013	.013	.013	-.007	.015	.007	.013	.003	.016	.033*	.016	-.011	.014	.009	.011	-.021	.015
Religiousity level	.002	.003	-.004	.003	.002	.003	-.003	.002	.001	.002	.004	.002	-.002	.002	.001	.002	-0.004*	.002	-.002	.002	-.003	.002	.006**	.002	.000	.002	.002	.001	.000	.002
Spirituality level	-.002	.003	-.001	.003	-.001	.002	-.001	.002	.001	.002	.000	.002	-.004*	.002	-.002	.002	.004*	.002	.000	.002	.001	.002	.000	.002	.003	.002	.002	.001	.001	.002

Appendix G: Results of 5 regressions (one for each musical feature group): Musical feature group frequency as a function of well-being goals (N=2,804) *p<0.1; **p<0.05; ***<p<0.01

	Volume		Mode		Tempo		Harmony		Timbre	
**Independent variables**	**B**	**SE**	**B**	**SE**	**B**	**SE**	**B**	**SE**	**B**	**SE**
Intercept	0.64***	.015	0.45***	.056	0.43***	.011	0.64***	.013	0.46***	.003
Release and venting of negative emotions	.014	.010	.024	.039	-.008	.008	-.001	.009	.002	.002
Diversion from the crisis	.014	.010	.026	.037	.012	.008	-.005	.009	-0.0034*	.002
Enjoyment and maintaining good mood	-.003	.011	-.037	.039	-.005	.008	-.006	.009	.001	.002
Reducing loneliness and creating a sense of "togetherness"	-.008	.010	.014	.039	.001	.008	.008	.009	.000	.002
Connecting with myself and detachment from the surroundings	-.012	.009	-.002	.034	.005	.007	.003	.008	.000	.002
Release and venting of negative emotions * music_importance	-.002	.002	-.008	.009	.003	.002	.001	.002	-.001	.000
Diversion from the crisis * music_importance	-.003	.002	-.008	.009	-.003	.002	.000	.002	.001	.000
Enjoyment and maintaining good mood * music_importance	.001	.003	.014	.009	.001	.002	.001	.002	.000	.000
Reducing loneliness and creating a sense of "togetherness" * music_importance	.001	.002	-.002	.009	-.001	.002	-.002	.002	.000	.000
Connecting with myself and detachment from the surroundings * music_importance	.002	.002	.001	.008	-.001	.002	-.002	.002	.000	.000
Age (25-44)	-.013	.007	.032	.025	.006	.005	-.003	.006	-.002	.001
Age (45-64)	-0.0469***	.009	.063	.035	-.007	.007	0.03**	.008	.000	.002
Age (over 64)	-0.0864***	.015	.051	.055	-0.0368**	.011	0.03*	.013	.000	.003
Gender (female)	.002	.006	0.0751***	.021	-.007	.004	0.02***	.005	-.001	.001
Gender (not to say)	-.017	.027	.052	.101	-.026	.021	.010	.024	.000	.005
Gender (self-describe)	-.007	.027	-.065	.099	.026	.020	-.005	.024	.003	.005
Personal status (in a relationship)	.004	.007	-.018	.027	.004	.005	.007	.006	.000	.001
Personal status (married/civil partnership)	.001	.008	.005	.031	.001	.006	.000	.008	-.002	.002
Personal status (divorced)	-.012	.015	.015	.055	.017	.011	-.022	.013	-.001	.003
Personal status (widowed)	-.005	.015	.047	.057	.000	.012	.019	.014	.002	.003
Personal status (other)	-.013	.023	-.066	.087	.002	.018	.006	.021	-.004	.004
Children (0)	.005	.009	-.057	.034	-.005	.007	.006	.008	.001	.002
Children (1)	.002	.009	.037	.035	.000	.007	0.017*	.008	-.001	.002
Children (2)	-.010	.014	.051	.052	-.009	.011	.006	.012	.004	.003
Children (3)	.020	.020	-.130	.073	.018	.015	-0.043*	.017	-.001	.004
Religiosity level	-.003	.003	.017	.010	-.003	.002	0.008***	.002	.001	.000
Spirituality level	-0.0065**	.002	-.003	.009	.000	.002	.000	.002	.000	.000
